# Effectiveness of a life story intervention for adults with intellectual disability and depressive and trauma‐related complaints

**DOI:** 10.1111/jar.12754

**Published:** 2020-06-15

**Authors:** Janny Beernink, Gerben J. Westerhof

**Affiliations:** ^1^ Dokter Bosman Mental Health Care Doetinchem The Netherlands; ^2^ Psychology, Health, and Technology University of Twente Enschede The Netherlands

**Keywords:** depressive complaints, intellectual disability, life story intervention, quisi‐experimental study, trauma‐related complaints

## Abstract

**Introduction:**

People with intellectual disability have a higher chance of developing mental disorders than the general population. Yet, few evidence‐based interventions exist. This article evaluates My Lifestory, a narrative intervention tailored to people with intellectual disability and depressive or trauma‐related complaints.

**Method:**

A quasi‐experimental research design was adopted with an experimental condition (My Lifestory) and a matched control condition (care as usual). Measurements took place before the intervention, at the end of the intervention and at follow‐up two months later. Measurements focused on psychiatric complaints, well‐being, life satisfaction, mastery, and purpose in life.

**Results:**

Participants in the intervention condition improved more in psychiatric complaints, well‐being, life satisfaction, and purpose in life, but not in mastery, than participants in the control condition. Effect sizes were large in the intervention condition and small in the control condition.

**Discussion:**

Despite some limitations, this study adds to the evidence base of this narrative intervention.

## 1 INTRODUCTION

Psychiatric symptoms are more common in people with (borderline) intellectual disability than in the general population (To, Neirynck, Vanderplasschen, Vanheule, & Vandevelde, 2014; Wieland, Aldenkamp, & van den Brink, 2017). This pertains in particular to depressive and trauma‐related symptoms (Kaatee, Troost, Jumelet, & Lindauer, 2016; Lakeman, Bodden, & Tromp, 2017; Wigham, Taylor, & Hatton, 2014). However, there are only a few well‐defined interventions specifically developed for people with intellectual disability and there is a scarcity of well‐conducted studies in clinical practice (Osugo & Cooper, 2016; Shepherd & Beail, 2017; Vlissides, Beail, Jackson, Williams, & Golding, 2017). Earlier work has mainly been conducted on cognitive‐behavioural therapy. Two systematic reviews conclude that existing studies on anxiety and depression show positive results, but that the evidence is still in an early, mainly descriptive phase (Dagnan, Jackson, & Eastlake, 2018; Jennings & Hewitt, 2015). There has also been some work on EMDR for post‐traumatic stress in people with intellectual disability (e.g. Mevissen, Didden, Korzilius, & De Jongh, 2017; see Gilderthorp, 2015 for a review). The current article describes the evaluation of *My Lifestory*, a narrative intervention that was specifically designed for persons with intellectual disability and psychiatric problems (Westerhof, Beernink, & Sools, 2016).

Life story interventions for persons with intellectual disability have attracted growing interest and appreciation in recent years, sometimes also in combination with mental health problems (e.g. Bunning, Gooch, & Johnson, 2017; Cloitre & Beck, 2017; Gonçalves, Machado, Martins, Hoek, & Machado, 2016; Grove, 2015; McParland, 2015). The self‐reported life stories of people with intellectual disability and psychiatric symptoms often pay little attention to their qualities, values, capabilities and talents. They are often saturated with painful events that are difficult to cope with: being bullied, experiencing otherness by exclusion from regular education and work, being dependent on special provisions, out‐of‐home placement, a lack of meaningful friendships, experiencing grief from typical loss events, experiences of failure and experiences of trauma or abuse (Marshall & Tilley, 2013; Wigham et al., 2014). In this way, they may be similar to stories of persons without intellectual disability, who also often present problem‐saturated stories in treatment (Westerhof & Bohlmeijer, 2012).

Life story interventions therefore focus on positive characteristics on the one hand, for example by retrieving specific positive memories, and on construing new meanings to negative events, for example by reconstructing the link between negative events and a person's identity (White & Epston, 1990). Earlier research in other groups, such as older persons with dementia or persons with depression, has shown that these kinds of interventions can be effective in both alleviating symptoms and promoting well‐being (Pinquart & Forstmeier, 2012; Westerhof & Slatman, 2019).

*My Lifestory* is an intervention that was specifically developed for persons with intellectual disability and depressive or trauma‐related symptoms (Westerhof et al., 2016). The design of the intervention followed guidelines for effective interventions for people with intellectual disability (de Wit, Moonen, & Douma, 2011): it paid special attention to limitations in working memory, executive functions and reflective power (Bai et al., 2014; Janssen & Schuengel, 2006). The intervention builds on the unique and personal life story and focuses on capabilities and strengths that can assist participants to identify and use coping strategies that are effective in minimizing the impact of painful situations or experiences. It follows the principles of narrative therapy in the deconstruction and reconstruction of personal stories (White & Epston, 1990) and the principles of life review therapy in the recollection, elaboration, evaluation and integration of stories from the past (Westerhof, 2019). Both painful and precious events are remembered because acknowledgement and confirmation of both storylines influence mental health (Korte, Bohlmeijer, Cappeliez, Smit, & Westerhof, 2012). By making thematic connections between past, present and future, participants are encouraged to discover what and who is of value in their lives. The intervention aims to decrease psychiatric symptoms and increase well‐being by developing rich storylines that provide space for identities beyond the identification with one's disability and psychiatric problems.

Previous studies showed that the intervention *My Lifestory* is well‐accepted by participants and practitioners and promising in decreasing psychiatric symptoms and increasing well‐being in persons with intellectual disability and depressive or trauma‐related symptoms (Beernink, Westerhof, & Sools, 2017; Westerhof et al., 2016). However, these studies used an observational design without a control group. The aim of the current study was to compare the effects of the intervention on psychiatric symptoms, well‐being, life satisfaction, mastery and purpose in life to a matched control group receiving care as usual. A secondary aim was to assess whether the effectiveness is different for people with different diagnoses (depression vs. post‐traumatic stress disorder) and different IQ levels (60–70 and 70–85).

## 2 METHOD

### 2.1 Design

The study took place in a Dutch psychiatric hospital, specialized in treating psychiatric disorders of persons with intellectual disability in inpatient and outreach programmes. The study was practice‐based, that is it followed everyday practices in the Dutch mental healthcare system with regard to the recruitment of the target group rather than recruiting persons for this intervention in an experimental setting. A quasi‐experimental research design was adopted with an experimental condition (participation in the intervention *My Lifestory*) and a control condition (participation in usual care). The participants in the control group were, as much as possible, matched on age, gender, diagnosis, IQ and setting (inpatient vs. outreach). Measurements took place before the intervention (baseline), immediately after the intervention (4 months after baseline) and at follow‐up (6 months after baseline).

### 2.2 Ethical approval

The study was approved by a Medical Ethics Committee (METiGG, nr. 08.151). Permission was requested from the participants themselves and/or their legal guardians on the basis of an adapted informed consent.

### 2.3 Conditions

Participants in the experimental condition followed the intervention *My Lifestory*. The present authors previously used the name *Who am I?*, but the content of the intervention is the same as described in detail in Westerhof et al. (2016). *My Lifestory* consists of 17 weekly structured one‐to‐one or group sessions, each lasting for 1.5–2 hr. The entire intervention spans a period of 4 months. It consists of three parts: past (nine sessions), present (six sessions) and future (two sessions). The past addresses themes such as birth, family, childhood, adolescence, education, and positive and negative life events. Participants use pictures, tell stories and acquire information from their family. The past is ritually closed by making a box with beautiful stories and one with difficult stories. The present addresses the current life phase, social contacts and identity and focuses on traits, talents, and values and norms. The future addresses a concrete life goal as well as a presentation and evaluation of what one has learned.

**Figure 1:**
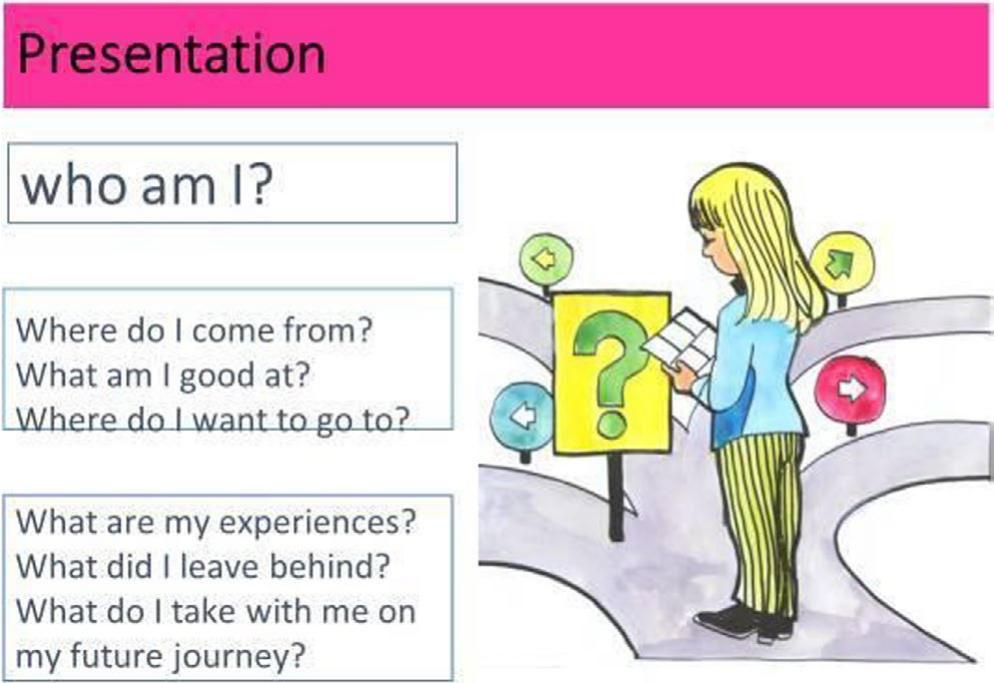
Example exercise from the intervention *My Lifestory*

The participants were invited to tell their life story from their own perspective based on a structured workbook (see Figure 1 for an example exercise). This workbook is written in accessible language with short sentences. Each of the three parts has its own colour. The intervention stays as close as possible to the experiences of the participants. It uses structured questions and assignments as well as metaphors and games to keep the intervention understandable and motivating. All areas of life are discussed to make the intervention broad and complete. It addresses the problems that persons with intellectual disability and depressive and trauma‐related symptoms might deal with, but also draws attention to competences, capabilities and talents. The intervention ends by the participants presenting their stories to people from their own social network, such as their parents, siblings or friends. The social network is also involved in the treatment to support participants in doing their homework, in using the (re)discovered qualities, strengths and skills in daily practice and in reducing the risk of relapse of psychiatric symptoms.

The intervention was guided by trained practitioners who were licensed healthcare psychologists. These practitioners followed a three‐day training programme in order to work with *My Lifestory,* that is also documented in a trainers manual (Beernink, 2018)*.* Practitioners were trained in the principles of life review and narrative therapy, in teaching the necessary communication skills, in handling strong emotions or other processing problems that may arise during the process, and in supporting participants to express feelings about their stories. They furthermore learned how to provide a safe and accepting environment, and how to use the manual and workbook. They practiced their competences through role‐playing and exercises. In the manual, the theory, the topics, the exercises, the materials and the home assignments are extensively described per session. Worksheets allow participants to write down their experiences for themselves. During the training, the practitioners carried out the intervention at their own workplace. At the end, when they successfully completed the training they received a certificate.

Participants in the intervention group received care as usual, such as cognitive‐behavioural therapy or problem‐solving therapy (once a week) or assertiveness training (once a week during 2 months). Those who were in the inpatient programme received daily individual contacts with social psychiatric nurses as well as daytime activities. Those who were in the outreach programme received weekly individual contacts with social psychiatric nurses.

### 2.4 Participants

Inclusion criteria for participating in the study were as follows: age 18 years and older, depression or post‐traumatic stress disorder (PTSS), and the willingness to be present and to do homework assignments. With regard to intellectual disability, a group with an IQ between 60 and 85 was targeted, that is around the DSM‐5 criterion for an IQ of 70, but also considered adaptive level, self‐reflection and self‐insight. An exclusion criterion was severe psychiatric disorders that made participation in a group unlikely. Licensed healthcare psychologists took the following tests: Beck Depression Inventory (BDI‐II; Beck, Steer, & Brown, 1996), Trauma Screening Questionnaire (TSQ; Brewin et al., 2002), Wechsler Adult Intelligence Scale (WAIS‐IV; Bouman, Hendriks, Kessels, & Aldenkamp, 2012) and a questionnaire for adaptive competences (AVVB; Kruisdijk, Jonker, Goedhard, & Nijman, 2019). All participants were diagnosed by a psychiatrist based on criteria of the DSM‐5.

A power analysis was conducted to estimate how many participants should be included in the study. To demonstrate a small effect size (*F* = 0.15) in a repeated‐measures ANOVA with two conditions, three measurements, a correlation between repeated measures of *r* = 0.60, a one‐sided alpha of 0.05 and a power of 0.80, a total sample size of 60 participants is required (G*Power). With a drop‐out rate of 25%, this means that 80 participants needed to be included.

Thirty‐eight participants with intellectual disability and depression or PTSS were recruited for the intervention condition. Forty participants with similar characteristics who received care as usual were recruited for the control condition. Matching was done as far as possible on gender, diagnosis, IQ, programme and age group. Thirty‐two participants completed the life story intervention My Lifestory. Three persons stopped due to personal circumstances such as moving house, two persons were discharged from the inpatient programme early, and one person was transferred to another ward. All participants who completed the life story intervention also completed all three assessments. Thirty participants in the control condition completed all assessments. For two participants, the care was stopped before the second measurement and eight participants did not want to fill out the questionnaires anymore after the first measurement.

Participants who completed all measurements did not differ significantly from those who did not in age, gender, diagnosis, IQ and location or in baseline measurements of symptoms, well‐being, life satisfaction, mastery or purpose in life. It was therefore decided to include only the participants who completed all assessments in the analyses.

Thirty‐two participants completed the life story intervention *My Lifestory,* and thirty participants comprised the control group. Table 1 summarizes the demographics of the participants. The groups did not differ on gender (61.3% female), age (54.9% between 18 and 44 years; 45.1% between 45 and 74 years), setting (48.4% inpatient; 51.6% outreach), diagnosis (58.1% depression and 41.9% trauma) or IQ range (60–70:37.1%; 70–85:62.9%).

**Table 1:** Demographics of participants

	Control group *N* = 30	Intervention group *N* = 32	All participants *N* = 62
Age			
18–24	2	5	7
25–34	7	6	13
35–44	6	7	13
45–54	8	11	19
55–64	6	3	9
65–74	1	0	1
Gender			
Male	12	13	25
Female	18	19	37
Setting			
Inpatient	18	18	36
Outreach	12	14	26
Diagnosis			
Trauma‐related symptoms	12	13	25
Depressive symptoms	18	19	37
Intelligence quotient			
60–70	12	10	22
70–85	18	22	40

### 2.5 Instruments

Five assessment instruments that were validated in the general population were used to collect data from all participants. Similar instruments showed adequate reliability and sensitivity to change in an earlier study on the intervention (Beernink et al., 2017; Westerhof et al., 2016), and other researchers also concluded that these kinds of instruments can be reliable and valid for people with intellectual disability (e.g. Kellett, Beail, Newman, & Mosley, 1999; Douma, 2018). A pedagogical employee who was not informed about the goals of the current study and who is trained in psychological testing supported participants, for example explaining difficult words, explaining and visualizing response alternatives, checking whether questions have been understood, avoiding distraction, inserting small breaks, allowing time to provide an answer and providing positive feedback on the commitment and attitude of the participant. All respondents were given a personal identification number, so the questionnaires were processed anonymously.

Psychiatric symptoms were assessed with the Outcome Questionnaire (OQ‐45; Lambert et al., 1999). The questionnaire measures symptomatic distress, interpersonal relations and social role. Participants rate how much they were bothered by 45 different symptoms for the past week on a 5‐point scale from “never” to “almost always.” The Cronbach alpha of the instrument is 0.93 in this study.

Well‐being was measured with the Dutch Mental Health Continuum‐Short Form (MHC‐SF; Lamers, Westerhof, Bohlmeijer, ten Klooster, & Keyes, 2011). The questionnaire measures three aspects of well‐being: emotional, psychological and social well‐being. Participants rate on 14 different items how often it applied to them during the past month on a 6‐point scale from “never” to “every day.” One question on social well‐being was not used because it was too difficult to understand for the target group. The reliability of the instrument is excellent in this study (Cronbach's alpha = 0.92).

The Satisfaction With Life Scale (SWLS; Van Beuningen, 2012; Pavot & Diener, 2008) measures life satisfaction. Participants rate five different items on a 5‐point scale from “totally not agree” to “totally agree.” The reliability of the instrument is good in this study (Cronbach alpha = 0.86).

The Mastery Scale (MS; Pearlin & Schooler, 1978) measures feelings of control in life. Participants rate how often they had thoughts on 5 different items on a 5‐point scale from “never” to “always.” The reliability (Cronbach's alpha) is 0.76 in this study.

Last, the Purpose in Life Scale (PIL; Ryff & Keyes, 1995) measures meaning in life. Participants rate how much they agree with five different items on a 5‐point scale from “totally not agree” to “totally agree.” In this study, the reliability of the instrument is 0.75 (Cronbach's alpha).

### 2.6 Analyses

Data were analysed by the second author using the Statistical Package for Social Sciences version 23. T tests were carried out to check whether the participants in the two conditions differed at baseline. For the main aim, repeated‐measures analyses of variance were performed in order to determine the changes in the experimental and the control condition across time. Condition was the independent variable, and the three measurement points were the dependent variables for each of the five measures separately. Simple contrasts were used to compare the effects after the intervention and at follow‐up with the baseline measurements. Next, paired effect sizes (Cohen's d) were calculated for the difference between the baseline and the follow‐up in each condition and computed the between‐group differences at follow‐up from these paired effect sizes. We interpreted effect sizes between 0.56 and 1.2 were interpreted as large, between 0.33 and 0.55 as moderate and below 0.33 as small (Lipsey & Wilson, 1993). For the secondary aim, diagnosis (depression version post‐traumatic stress disorder) was added to the repeated‐measures ANOVA, making it a 2 (depression versus trauma) by 2 (intervention versus control) by 3 (measurement points) analysis. A similar 2 × 2 × 3 repeated‐measures ANOVA was carried out with IQ (60–70 vs. 70–85) as independent variable.

## 3 RESULTS

First, the differences at baseline were analysed. There were no significant differences for psychiatric symptoms, well‐being, mastery and purpose in life (all *t* tests with *p *> .05), but participants in the control condition started with a higher life satisfaction (mean = 15.6; *SD* = 5.2) than participants in the intervention condition (mean = 12.8; *SD* = 5.5; *t*_60_ = 2.1; *p* = .042).

The results of the repeated‐measures ANOVA to assess the main aim are reported in Table 2. The main effects of condition are not significant. The differences of time are significant for all five measures, indicating that across conditions, participants decreased in psychiatric symptoms and increased in well‐being, life satisfaction, mastery and purpose in life. The interaction effects showed that the decrease in psychiatric symptoms is significantly stronger in the intervention condition than in the control condition. Furthermore, the increase in well‐being, life satisfaction and purpose in life is significantly stronger in the intervention than the control condition. The post hoc analyses showed that these interaction effects exist directly after the intervention (4 months; *p *< .05) as well as at follow‐up (2 months after the end of the intervention; *p *<. 05). The between‐group effect sizes at follow‐up were moderate for psychiatric symptoms (Cohen's *d* = 0.51); well‐being (Cohen's *d* = 0.57); life satisfaction (Cohen's *d* = 0.62); and purpose in life (Cohen's *d* = 0.46). There was no effect for mastery (Cohen's *d* = .‐0.02).

**Table 2:** Changes in outcome measures (*N* = 62)

	Experimental condition		Control condition		Condition	Time	Condition*Time
	Mean	*SD*	Mean	*SD*	*F*(1,60)	*F*(2,60)	*F*(2,60)
Psychiatric symptoms							
Baseline	76.0	28.6	65.2	25.4	0.0	12.3*	3.9*
4 months	57.2	23.3	61.0	29.8			
6 months	54.2	25.1	58.2	21.7			
Well‐being							
Baseline	2.1	1.3	2.6	1.2	0.0	7.4*	3.1*
4 months	2.9	1.2	2.8	1.3			
6 months	3.0	1.1	2.8	1.0			
Life satisfaction							
Baseline	2.6	1.1	3.1	1.0	0.6	7.1*	4.5*
4 months	3.2	0.9	3.1	1.0			
6 months	3.3	0.8	3.2	0.8			
Mastery							
Baseline	1.9	0.7	2.0	0.7	0.0	10.7*	1.0
4 months	2.3	0.6	2.2	0.8			
6 months	2.3	0.8	2.4	0.7			
Purpose in life							
Baseline	3.1	0.9	3.2	1.1	1.7	8.4*	4.6*
4 months	3.9	1.0	3.3	1.0			
6 months	3.7	0.9	3.4	0.8			

For the second aim, diagnosis (depression versus PTSS; *N* = 36 and 26) was added as independent variable to the repeated‐measures analysis. There were no significant interactions between diagnosis, time and condition (F_psychiatric symptoms_ = 0.3; F_well‐being_ = 0.1; F_life satisfaction_ = 0.0; F_mastery_ = 0.3; F_purpose in life_ = 0.9; all *p*>.05). In a separate analysis, IQ (60–70 versus 70–85; *N* = 23 and 39) was added to the repeated‐measures analysis. Again, there were no significant interactions between IQ, time and condition (F_psychiatric symptoms_ = 0.6; F_well‐being_ = 1.0; F_life satisfaction_ = 0.4; F_mastery_ = 0.6; F_purpose in life_ = 1.1; all *p *> .05). Hence, the stronger effects in the intervention condition apply to both diagnostic groups and people with higher and lower IQ.

## 4 DISCUSSION

The present study evaluated the effectiveness of *My Lifestory* for people with intellectual disability and depressive or trauma‐related symptoms in comparison with a matched control group that received care as usual. With regard to the main aim, the results of the study showed that participants in the intervention improved more in psychiatric symptoms, well‐being, life satisfaction and purpose in life, but not in mastery in comparison with participants who received care as usual. The differences in improvement are moderate when comparing the intervention condition to the control condition. With regard to the secondary aim, no differences were found for participants with depression versus PTSS or with lower versus higher IQ.

The results of the current study are in line with earlier studies on this intervention that did not use a control group, but reported similar improvements in psychiatric symptoms and well‐being (Beernink et al., 2017; Westerhof et al., 2016). In both studies, there were similar changes in mastery and purpose in life, but these were significant for mastery in the previous study (Beernink et al., 2017) and for purpose in life in the current study, probably due to the fact that more participants and a control group were included in the present study. The results are also in line with a meta‐analysis on life review interventions in older individuals without intellectual disability. This meta‐analysis found small‐to‐moderate effects of life review in later life on depressive symptoms, well‐being, mastery and purpose in life but did not include life satisfaction (Pinquart & Forstmeier, 2012). The current study contributes to the evidence base of narrative interventions for people with intellectual disability that included fewer participants or no control group (e.g. Bunning et al., 2017; Cloitre & Beck, 2017; Fredman, 2014; Gonçalves et al., 2016; Grove, 2015; McParland, 2015; Peterson, Gillam, Spencer, & Gillam, 2010). It also adds to the evidence base of well‐defined interventions specifically developed for people with intellectual disability and psychiatric symptoms (Ali, Hall, Blickwedel, & Hassiotis, 2015; Kok, van der Waa, Klip, & Staal, 2016; McCabe, McGillivray, & Newton, 2006; Osugo & Cooper, 2016; Shepherd & Beail, 2017; Vlissides et al., 2017).

A number of limitations of the current study should be noted. First, the current study took place in a practice context where it is not allowed to have persons wait for treatment for half a year. A matched control group was therefore used. Further research is needed to assess whether the stronger changes can indeed be attributed to the intervention. A randomized controlled trial that fulfils all quality criteria including issues such as masking, allocation concealment, independent data management and assessment of treatment fidelity is needed (Higgins et al., 2011). An interesting and cost‐effective alternative in this clinical context is a multiple baseline single‐case study that tracks a number of participants intensively before, during and after the intervention (Kazdin, 2019). Second, the intervention consists of many components. For example, the involvement of the participant's network might also have contributed to the effects. Further research should clarify which of these components are necessary to decrease symptoms and promote well‐being. Third, only participants who completed all assessments were included. Although this might have led to an overestimation of effects, there were no significant differences in background characteristics or baseline assessments between participants in the intervention group who did or did not complete all measures. Fourth, the follow‐up two months after the end of the 17‐week intervention is rather short. Fifth, the study only made use of self‐report measures. Participants with lower IQ levels might have had difficulty to fill out the instruments, but they were supported by trained staff to do so. The finding that not all instruments showed similar effects suggests that participants did discriminate between the instruments while providing their answers. Other studies also found that people with intellectual disability are able to provide reliable and valid answers to self‐report instruments (Douma, Dekker, Verhulst, & Koot, 2006; Hurley, 2008). The intervention followed current procedures in mental health care and was delivered in everyday practice. This might imply that it was not always delivered as intended. Therapy fidelity was not assessed, but the clinicians were trained in the life story intervention. It would be of interest to assess how the intervention was delivered and how participants told their stories, for example by observations and analyses of products.

Despite these limitations, the current study contributes further evidence that participants may profit from the life story intervention in terms of reducing their symptoms and increasing their well‐being. This is important, as few interventions have been evaluated that were specifically developed for persons with intellectual disability. The study has important clinical implications. Traditional mental health care often does not have interventions that are tailored to persons with intellectual disability, whereas traditional care for persons with intellectual disability often does not have the expertise to treat their mental health problems such as depression or trauma. The current intervention addresses this gap. For further implementation, a train‐the‐trainer, a manual for practitioners and a workbook for participants have been published in Dutch (Beernink, 2018). However, the intervention has now been guided by licensed healthcare psychologists in inpatient and outreach settings in the Netherlands with a particular focus on this group with both intellectual and psychiatric challenges. It is important to address questions regarding implementation in the separate fields of care for mental health and intellectual disability in other countries as well. Furthermore, more research is needed on the necessary length of the intervention and the necessary qualifications as trained and licensed healthcare psychologists are not always available.
